# Post-treatment serum triglyceride: An effective biomarker for body fat mass and overall survival in esophageal squamous cell cancer patients treated with chemoradiotherapy

**DOI:** 10.3389/fnut.2022.1050643

**Published:** 2022-12-02

**Authors:** Jiahua Lyu, Ningjing Yang, Wang Guan, Ling Xiao, Xinyu Nie, Long Liang, Hansong Bai, Churong Li, Hao Kuang, Xiao Wang, Tao Li

**Affiliations:** ^1^School of Medicine, University of Electronic Science and Technology of China, Chengdu, China; ^2^Sichuan Cancer Hospital, School of Medicine, University of Electronic Science and Technology of China, Chengdu, China

**Keywords:** serum triglyceride, esophageal squamous cell carcinoma, body fat mass, overall survival, biomarker

## Abstract

**Objectives:**

Although lipids have been assessed for their possible roles in cancer survival prediction, studies on the association between serum triglyceride (TG) levels and the prognosis of esophageal squamous cell carcinoma (ESCC) patients are limited. This study aimed to evaluate whether serum TG is associated with outcomes in patients with ESCC and investigate any interaction between serum TG and clinical parameters, especially body fat mass.

**Materials and methods:**

We conducted a prospective case study on patients diagnosed with ESCC between March 2012 and November 2018. We measured patients’ serum TG levels before and after treatment. The association between serum TG and overall survival (OS) was evaluated using hazard ratios. We sought to determine a threshold point using optimal stratification. Survival analysis was performed using Kaplan–Meier curves and a Cox proportional hazards model.

**Results:**

Of the 257 participants diagnosed with ESCC, 200 (77.8%) were men. Median follow-up time was 22.4 months (range 3.3–92.4 months). Using univariate Cox proportional hazard analysis and subsequent multivariate analysis, post-TG levels, Karnofsky performance scores, T stages, and chemotherapy cycles were shown to be independent prognostic factors for OS (*p* < 0.05). The post-TG cut-off point to best classify patients with respect to time to mortality was 1.47 mmol/L. A post-TG level of ≥ 1.47 mmol/L could independently predict a better OS (hazard ratio: 0.55, 95% confidence interval: 0.37–0.79). The associations were consistent across the subtypes of clinical parameters. Furthermore, the post-body mass index, post-subcutaneous adipose tissue area, post-visceral adipose tissue area, post-total adiposity tissue area, and post-total adipose density exhibited a strong positive association with post-TG levels.

**Conclusion:**

Post-TG levels were found to be a significant positive prognostic biomarker for body fat mass and OS in ESCC patients.

## Introduction

Esophageal cancer is ranked as the eighth leading cause of cancer-associated deaths worldwide, and approximately 70% of global esophageal cancers occur in China, where the major histologic type is esophageal squamous cell cancer (ESCC) (1). Although concurrent chemoradiotherapy (CCRT) is currently accepted as the standard therapy for patients with locally advanced ESCC, the average 5-years survival rate is less than 30% (2). Therefore, finding effective, inexpensive, and accurate biomarkers for prognosis evaluation and thereby helping tailor therapies for ESCC patients can be extremely helpful in the clinic.

Triglyceride (TG) is an important component of blood lipids, which comes from decomposition of fat in food or conversion from fructose in the liver. For a long time, TG has been regarded as a nutritional index, which is closely related to obesity and the occurrence of cardiovascular diseases (3–6). In recent years, more and more studies have been conducted to shed light on the relationship between TG and tumorigenesis and prognosis of cancer patients. TG has been proven to be associated with cancer cell proliferation and tumor growth (7, 8). Several investigations have indicated that serum TG levels are a risk factor for cancers (9, 10).

Furthermore, the relationship between serum TG levels and cancer prognosis of various cancers has been studied. Li et al. showed that pre-operative lower triglycerides were risk factors for breast cancer patients. Patients with low level TG showed remarkably worse OS (*P* = 0.078) and DFS (*P* = 0.008) than high level (11). Hu and Sun et al. both observed that preoperative serum TG levels were a powerful predictors of postoperative gastric cancer mortality (12, 13).

As is well-known, serum TG is largely affected by diet and nutritional status. Meanwhile, poor diet intake and malnutrition are common comorbidities and vital factors that affect prognosis in patients with esophageal cancer. Therefore, theoretically, serum TG may be correlated with the prognosis of patients with esophageal cancer, but there is insufficient clinical evidence. To date, only one study have explored the association between serum TG levels and survival in esophageal cancer patients, which demonstrated that prognostic nomogram based on pretreatment serum lipids could be applied to predict OS and DFS in non-ESCC patients (14).

Thus, in this study, our objective was to determine whether TG levels before and after treatment (pre-TG and post-TG) are associated with outcomes for patients with ESCC and establish a cut-off point for low and high TG levels based on a Chinese population. In addition, we investigated whether there was any significant association between serum TG and patients’ clinical parameters, especially body fat mass.

## Materials and methods

### Study population

The eligibility criteria for the current study were as follows: patients aged > 18 years, ESCC confirmed by a histological or cytological diagnosis, patients who received curative-intent radiotherapy, and patients with complete laboratory and clinical data. Participants were excluded according to the following criteria: operable ESCC, use of drugs affecting lipid metabolism, and any other primary tumor in addition to esophageal cancer. Approval for this trial was obtained from the Ethics Committee and the Institutional Review Board of Sichuan Cancer Hospital. Finally, 257 patients with ESCC (stages II–IVA) confirmed by histopathology, diagnosed between March 2012 and November 2018 at the Cancer Center of were included in this study.

### Clinical parameters and laboratory data

Patient data including demographic parameters, clinical parameters, and laboratory data were collected from the electronic medical records database, including information on sex, age, smoking history, alcohol consumption history, T stage, N stage, Karnofsky performance score, tumor location, tumor length, height, body weight, radiotherapy dose, chemotherapy drugs, chemotherapy cycles, blood glucose, and blood lipid levels. Blood samples were collected on an empty stomach within 3 days of the CT scan and analyzed by enzymatic colorimetric methods using an automatic biochemical analyzer (Mindray BS2000M; Shenzhen Mindray Bio-Medical Electronics Co., Ltd., Shenzhen, China).

### Body composition assessment

The area and radiodensity of muscle and adipose tissue were measured using computed tomography before and after treatment. Two experienced radiologists used SliceOmatic Software version 5.0 (TomoVision) for calculating the cross-sectional area of the muscle and adipose tissue in centimeters squared at the third lumbar vertebra (L3) (15). Muscle area (MA), muscle density (MD), visceral adipose tissue area (VAA), subcutaneous adipose tissue area (SAA), and total adipose density (TAD) were quantified separately. Total adiposity tissue area (TAA) was calculated as the sum of VAA and SAA.

### Follow-up

Patients were regularly followed-up after treatment at 3-month and 6-month intervals for the initial 2 years and the next 3–5 years subsequently. Overall survival (OS) was calculated as the number of days from the date of ESCC diagnosis to the date of last contact or death.

### Statistical analysis

The R software, version 4.0.2 (R Foundation for Statistical Computing), was used to perform all statistical analyses in the current study. Descriptive statistics were shown as mean ± standard deviation, while median (interquartile range) or frequencies (percentages) were used to describe the skewed distribution data. Chi-square test or Fisher’s exact test was used to test the difference of categorical variables between patients alive and dead. Mann-Whitney *U* test or Student *t*-test was used to test the difference of continuous variables between patients alive and dead. Pearson’s correlation analysis was used to test the association between TG levels and body composition. Univariate and multivariate regression analyses were conducted using the Cox proportional hazards model to evaluate the association between TG and OS. The Schoenfeld residuals were used to test the PH assumption (16). We performed a multivariate regression analyses employing a stepwise selection method. An outcome-oriented method was used to determine the optimal cut-off point for continuous variables to maximize log-rank statistics. To test if there was any association between TG levels and various patients’ parameters, interaction terms were used. All statistical tests performed were two-tailed, and *p*-values < 0.05 were considered statistically different.

## Results

### Patients with esophageal squamous cell carcinoma had a higher mean triglyceride level after treatment than before treatment

**FIGURE 1:**
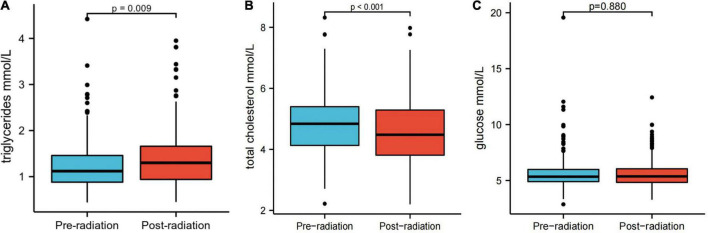
Serum triglyceride **(A)**, total cholesterol **(B)**, and glucose levels **(C)** before and after treatment.

Serum TG, total cholesterol (TC), and glucose levels were compared between patients before and after treatment. Notably, ESCC patients exhibited significantly higher mean TG (1.30 mmol/L vs. 1.12 mmol/L, *p* = 0.009) and lower mean TC levels after treatment than before treatment (4.48 mmol/L vs. 4.84 mmol/L, *p* < 0.001) (Figure 1). There was no significant difference in serum glucose levels before and after treatment (Figure 1).

### Association of serum triglyceride with overall survival in esophageal squamous cell carcinoma patients

Median follow-up time was 22.4 months (range 3.3–92.4 months). Until the last follow-up, there were 97 patients alive, whereas 160 patients were deceased. The distribution of background variables stratified by survival status is presented in Table 1. Notably, patients alive exhibited significantly a KPS of 90 or greater, early T stage, early N stage, higher pre-TC, higher post-TC, higher post-TG and shorter tumor length compared with those who died (*p* < 0.05).

**TABLE 1:** The distribution of background variables stratified by survival status.

Characteristic	All patients (*N* = 257)	Alive (*N* = 97)	Death (*N* = 160)	*P*-value
Sex, n (%)				1.000
Female	57 (22.2%)	22 (22.7%)	35 (21.9%)	
Male	200 (77.8%)	75 (77.3%)	125 (78.1%)	
KPS, n (%)				**0.027**
≤ 80	114 (44.4%)	34 (35.1%)	80 (50.0%)	
≥ 90	143 (55.6%)	63 (64.9%)	80 (50.0%)	
Smoking history, n (%)				0.798
No	110 (42.8%)	43 (44.3%)	67 (41.9%)	
Yes	147 (57.2%)	54 (55.7%)	93 (58.1%)	
Alcohol history, n (%)				0.262
No	117 (45.5%)	49 (50.5%)	68 (42.5%)	
Yes	140 (54.5%)	48 (49.5%)	92 (57.5%)	
Tumor location, n (%)				0.484
Cervical	8 (3.1%)	5 (5.2%)	3 (1.9%)	
Upper thoracic	53 (20.6%)	22 (22.7%)	31 (19.4%)	
Middle thoracic	96 (37.4%)	34 (35.1%)	62 (38.8%)	
Lower thoracic	88 (34.2%)	33 (34%)	55 (34.4%)	
Abdominal	12 (4.7%)	3 (3.1%)	9 (5.6%)	
T stage, n (%)				**<0.001**
T2	29 (11.3%)	24 (24.7%)	5 (3.1%)	
T3	114 (44.4%)	50 (51.5%)	64 (40.0%)	
T4	114 (44.4%)	23 (23.7%)	91 (56.9%)	
N stage, n (%)				**0.002**
N0	7 (2.7%)	6 (6.2%)	1 (0.6%)	
N1	80 (31.1%)	30 (30.9%)	50 (31.2%)	
N2	118 (45.9%)	50 (51.5%)	68 (42.5%)	
N3	52 (20.2%)	11 (11.3%)	41 (25.6%)	
Chemotherapy cycle, n (%)				0.397
0	39 (14.8%)	11 (11.3%)	28 (17.5%)	
1	25 (9.7%)	6 (6.2%)	19 (11.9%)	
2	93 (36.2%)	39 (40.2%)	54 (33.8%)	
3	56 (21.8%)	23 (23.7%)	33 (20.6%)	
4	40 (15.6%)	16 (16.5%)	24 (15%)	
5	3 (1.2%)	1 (1%)	2 (1.2%)	
6	1 (0.4%)	1 (1%)	0 (0%)	
Chemotherapy drugs, n (%)				0.189
PF	80 (36.9%)	27 (31.0%)	53 (40.8%)	
TP	137 (63.1%)	60 (69.0%)	77 (59.2%)	
Pre-glucose median (IQR)	5.34 (4.90, 5.99)	5.40 (4.95, 5.92)	5.29 (4.85, 6.04)	0.799
Pre-TC median (IQR)	4.84 (4.13, 5.40)	5.11 (4.45, 5.74)	4.67 (4.06, 5.26)	**0.003**
Pre-TG median (IQR)	1.12 (0.88, 1.46)	1.12 (0.87, 1.5)	1.12 (0.88, 1.43)	0.524
Post-glucose median (IQR)	5.37 (4.83, 6.05)	5.37 (4.83, 6.05)	5.37 (4.83, 6.04)	0.982
Post-TC median (IQR)	4.48 (3.81, 5.29)	4.91 (4.08, 5.52)	4.21 (3.71, 5.14)	**0.001**
Post-TG median (IQR)	1.30 (0.94, 1.66)	1.45 (1.03, 1.83)	1.19 (0.9, 1.49)	**<0.001**
Age, mean ± SD	64.0 ± 8.72	63.4 ± 7.71	64.03 ± 10.28	0.578
Tumor length, median (IQR)	5 (4, 7)	5 (3, 6)	5 (4, 7)	**0.016**

KPS, karnofsky performance score; PF, 5-Fluorouracil + cisplatin; TP, paclitaxel + cisplatin; TC, total cholesterol; TG, triglyceride; IQR, Inter quartile range; SD, stand arddeviation. Bold values represent the statistical differences.

Univariate and multivariate Cox regression analyses were used to determine the risk factors for OS (Table 2). Univariate analysis of clinical features revealed that tumor length, T stage, N stage, chemotherapy cycle, pre-TC, post-TC, and post-TG were significantly associated with survival (*p* < 0.05). Multivariate analysis revealed that Karnofsky performance score, T stage, and post-TG were independent prognostic factors. We then tested the PH assumption and found that the *p*-values for all three individual variables (KPS, T stage, and post-TG) were greater than 0.05, indicating that each variable satisfied the PH assumption, while the overall test of the model had a *p*-value of 0.536 and the model as a whole satisfied the PH assumption (Supplementary Figure 1).

**TABLE 2:** Univariate and multivariate Cox regression analyses of factors associated with overall survival.

Characteristics	Total (*N*)	Univariate analysis		Multivariate analysis	
		Hazard ratio (95% CI)	*P*-value	Hazard ratio (95% CI)	*P*-value
Sex	257				
Female	57	Reference			
Male	200	0.99 (0.68–1.45)	0.985	1.20 (0.79–1.83)	0.381
Age	257	1.01 (0.99–1.03)	0.230	0.99 (0.97–1.02)	0.605
KPS	257	0.97 (0.95–1.00)	0.071	0.97 (0.94–0.99)	**0.029**
Smoking history	257				
No	110	Reference			
Yes	147	1.11 (0.81–1.52)	0.524		
Alcohol history	257				
No	117	Reference			
Yes	140	1.28 (0.94–1.75)	0.122		
Tumor location	257				
Cervical	8	Reference			
Upper thoracic	53	2.19 (0.67–7.16)	0.196		
Middle thoracic	96	2.30 (0.72–7.32)	0.160		
Lower thoracic	88	2.11 (0.66–6.77)	0.207		
Abdominal	12	2.46 (0.67–9.11)	0.177		
Tumor length	257	1.08 (1.02—1.15)	**0.010**		
T stage	257				
T2	29	Reference			
T3	114	5.16 (2.07–12.88)	**<0.001**	2.43 (1.64–3.61)	**<0.001**
T4	114	15.45 (6.13–38.93)	**<0.001**	14.09 (5.44–37.04)	**<0.001**
N stage	257				
N0	7	Reference			
N1	80	6.66 (0.92–48.25)	0.061	4.19 (0.51–34.79)	0.184
N2	118	6.55 (0.91–47.21)	0.062	3.96 (0.48–32.76)	0.202
N3	52	15.84 (2.17–115.63)	**0.006**	4.83 (0.57–41.14)	0.149
Chemotherapy cycle	257	0.83 (0.73–0.94)	**0.003**	0.89 (0.75–1.07)	0.218
Chemotherapy drugs	217				
PF	80	Reference			
TP	137	0.76 (0.54–1.08)	0.129	0.84 (0.45–1.55)	0.571
Pre-glucose	257	0.89 (0.78–1.01)	0.073	0.95 (0.81–1.12)	0.539
Pre-TC	257	0.79 (0.67–0.93)	**0.005**	0.85 (0.69–1.05)	0.125
Pre-TG	257	0.87 (0.64–1.18)	0.355	0.76 (0.54–1.06)	0.109
Post-glucose	257	0.96 (0.84–1.10)	0.541	0.97 (0.81–1.15)	0.690
Post-TC	257	0.79 (0.67–0.92)	**0.002**	0.93 (0.77–1.12)	0.452
Post-TG	257	0.56 (0.42–0.76)	**<0.001**	0.61 (0.42–0.89)	**0.009**

KPS, karnofsky performance score; PF, 5-Fluorouracil + cisplatin; TP, paclitaxel + cisplatin; TC, total cholesterol; TG, triglyceride. Bold values represent the statistical differences.

Nomograms were constructed to predict OS at 2, 3, and 4 years (Supplementary Figure 2A). The CI for our survival model was 0.73 (95% confidence interval: 0.71–0.74), which showed good agreement between the survival predicted by the nomogram and the actual survival. The calibration curve showed that this nomogram exhibited good calibration (Supplementary Figure 2B).

We analyzed the change between pre- and post-treatment TG as a continuous variable in univariate and multivariate analysis, and found that the change of TG is also a prognostic indicator of survival (HR 0.72, 95% CI 0.55–0.94, *p* = 0.015), but not as well as the post-TG (HR 0.61, 95% CI 0.42–0.89, *p* = 0.009) (Supplementary Table 1).

### Patient demographics and disease characteristics stratified by post-triglyceride

**FIGURE 2:**
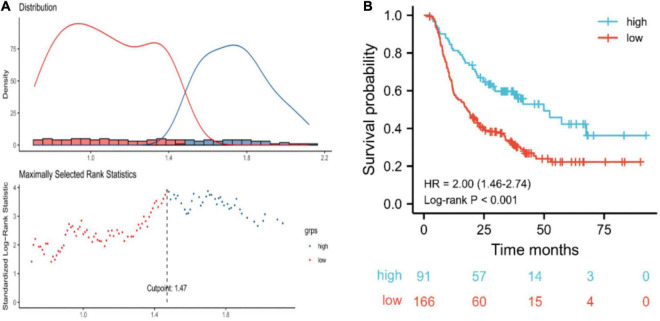
**(A)** Optimal cut-off points were determined using an outcome-oriented method. **(B)** Overall survival of esophageal squamous cell carcinoma patients based on post-triglyceride (TG) cut-off. For post-TG, low: < 1.47 mmol/L; high: ≥ 1.47 mmol/L.

The cut-off for post-TG associated with OS was 1.47 mmol/L (Figure 2). Based on the post-TG cut-off, 91 ESCC patients were categorized into the high post-TG group (> 1.47 mmol/L) and 166 in the low post-TG group (< 1.47 mmol/L). Kaplan–Meier curves and log-rank test results indicated that the high post-TG group exhibited a better prognosis than the low post-TG group (Figure 2).

The results of multivariate Cox regression models for post-TG and OS are shown in Table 3. Post-TG was positively related to better prognosis after adjusting for sex, age, Karnofsky performance score, smoking consumption, alcohol consumption, tumor length, tumor location, T stage, N stage, chemotherapy cycle, chemotherapy drugs, and pre-glucose, pre-TC, pre-TG, post-glucose, and post-TC levels.

**TABLE 3:** Hazard risk for mortality in patients with high post-triglyceride (TG).

Model	Characteristic	HR (95% CI)	*P*-value
Model A	Low (< 1.47)	Ref	
	High (≥ 1.47)	0.51 (0.36–0.72)	<0.001
Model B	Low (< 1.47)	Ref	
	High (≥ 1.47)	0.50 (0.35–0.72)	<0.001
Model C	Low (< 1.47)	Ref	
	High (≥ 1.47)	0.54 (0.37–0.79)	0.001

TG, triglyceride; CI, confidence interval; HR, hazard ratio. Model A was adjusted for age, sex, Karnofsky performance score, smoking, and alcohol consumption. Model B was adjusted for Model 1 plus tumor location, tumor length, T stage, N stage, chemotherapy cycle, and chemotherapy drugs. Model C was adjusted for Model B plus pre-glucose, pre-total cholesterol, pre-TG, post-glucose, and post-total cholesterol levels.

### Stratified analyses performed by patient characteristics

**FIGURE 3:**
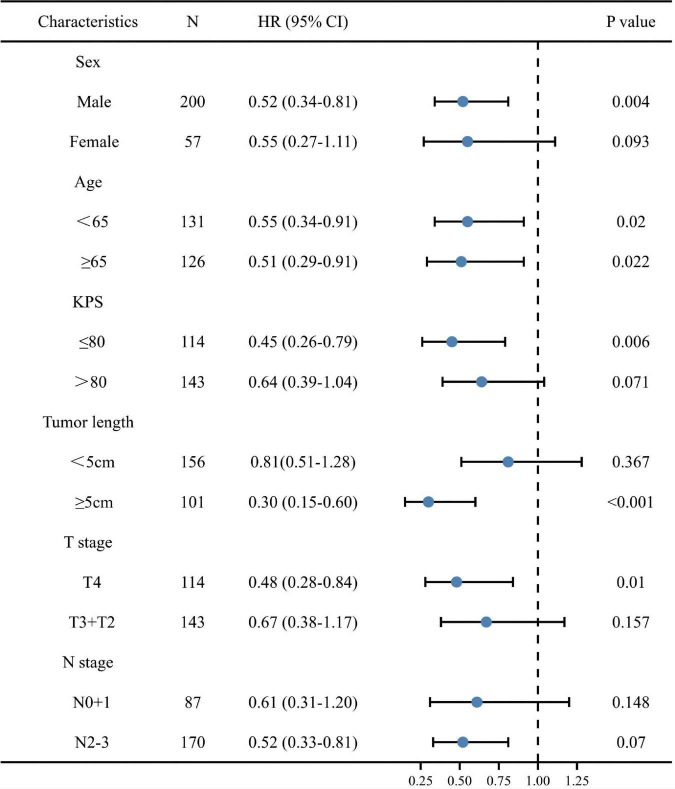
A forest plot of overall survival (OS) for various patient subgroups of esophageal squamous cell carcinoma. CI, confidence interval; HR, hazard ratio; KPS, Karnofsky performance score.

We performed stratified analyses to clarify the relationship between post-TG and the hazard ratio of OS in subgroups (Figure 3). Overall, high post-TG was consistently associated with a decreased mortality risk across each subgroup of ESCC patients, except for patients with early T stage (T2 + T3), early N stage (N0 + 1) and tumor length < 5 cm (*p* < 0.10).

Table 4 presents a comparison of the patients’ characteristics between the low and high post-TG groups.

**TABLE 4:** Demographic and clinical characteristics of cancer patients stratified by post-triglyceride (TG).

Characteristic	High post-TG	Low post-TG	*P*-value
N	91	166	
Sex, n (%)			0.597
Female	18 (7.0%)	39 (15.2%)	
Male	73 (28.4%)	127 (49.4%)	
KPS, n (%)			0.535
70	1 (0.4%)	5 (1.9%)	
80	42 (16.3%)	66 (25.7%)	
90	48 (18.7%)	93 (36.2%)	
100	0 (0%)	2 (0.8%)	
Smoking history, n (%)			0.885
No	40 (15.6%)	70 (27.2%)	
Yes	51 (19.8%)	96 (37.4%)	
Alcohol history, n (%)			0.587
No	44 (17.1%)	73 (28.4%)	
Yes	47 (18.3%)	93 (36.2%)	
Tumor location, n (%)			0.179
Cervical	3 (1.2%)	5 (1.9%)	
Upper thoracic	22 (8.6%)	31 (12.1%)	
Middle thoracic	25 (9.7%)	71 (27.6%)	
Lower thoracic	37 (14.4%)	51 (19.8%)	
Abdominal	4 (1.6%)	8 (3.1%)	
T stage, n (%)			0.472
T2	9 (3.5%)	20 (7.8%)	
T3	37 (14.4%)	77 (30%)	
T4	45 (17.5%)	69 (26.8%)	
N stage, n (%)			0.388
N0	2 (0.8%)	5 (1.9%)	
N1	23 (8.9%)	57 (22.2%)	
N2	44 (17.1%)	74 (28.8%)	
N3	22 (8.6%)	30 (11.7%)	
Chemotherapy cycle, n (%)			0.466
0	15 (5.8%)	24 (9.3%)	
1	5 (1.9%)	20 (7.8%)	
2	37 (14.4%)	56 (21.8%)	
3	18 (7.0%)	38 (14.8%)	
4	14 (5.4%)	26 (10.1%)	
5	2 (0.8%)	1 (0.4%)	
6	0 (0.0%)	1 (0.4%)	
Chemotherapy drugs, n (%)			0.056
PF	35 (16.1%)	45 (20.7%)	
TP	41 (18.9%)	96 (44.2%)	
Pre-glucose, median (IQR)	5.35 (4.91, 6.02)	5.31 (4.89, 5.94)	0.859
Pre-TC, median (IQR)	4.9 (4.17, 5.40)	4.83 (4.08, 5.39)	0.759
Pre-TG, median (IQR)	1.14 (0.91, 1.41)	1.12 (0.86, 1.52)	0.708
Post-glucose, median (IQR)	5.35 (4.88, 6.18)	5.37 (4.8, 6.04)	0.934
Post-TC, median (IQR)	4.22 (3.74, 5.10)	4.53 (3.88, 5.32)	0.155
Age, mean ± SD	63.3 ± 9.07	64.07 ± 9.57	0.531
Tumor length, median (IQR)	5 (4.0, 7.0)	5 (3.1, 7.0)	0.047

KPS, karnofsky performance score; PF, 5-Fluorouracil + cisplatin; TP, paclitaxel + cisplatin; TC, total cholesterol; TG, triglyceride.

### Associations between post-triglyceride and body composition

**FIGURE 4:**
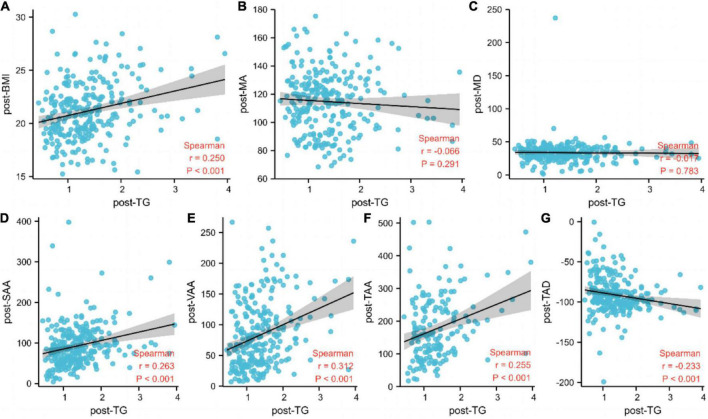
Associations between post-triglyceride and body composition. **(A)** BMI, body mass index; **(B)** MA, muscle area; **(C)** MD, muscle density; **(D)** SAA, subcutaneous adipose tissue area; **(E)** VAA, visceral (INTRA–ABDOMINAL) adipose tissue area; **(F)** TAA, total adiposity tissue area; **(G)** TAD, total adipose density; TG, triglyceride.

Next, we analyzed the associations between body composition assessment, including muscle area and radiodensity, and adiposity with post-TG. The post-body mass index, post-subcutaneous adipose tissue area, post-visceral (intra-abdominal) adipose tissue area, post-total adiposity tissue area, and post-total adipose density exhibited a strong positive association with post-TG (Figure 4).

## Discussion

Dyslipidemia is associated with tumor formation and development, and serum lipid levels can be used as a cancer biomarker for prognosis prediction/patient stratification. TG is one of the main constituents of blood lipids and energy reservoirs (17). Several studies have reported an association between TG and prognosis in cancer patients. A retrospective review of 551 NSCLC patients revealed that a high preoperative TG level was associated with poor OS (18). Another retrospective study, including stage IB1-IIA2 cervical cancer patients, also showed that the high TG group was associated with worse OS (19). Chen et al. conducted a study that included 246 high-risk stage II and stage III colorectal cancer patients undergoing radical surgery. In the study, multivariate analysis demonstrated that high TG was significantly associated with decreased disease-free survival (DFS) and OS (20). However, in contrast to the cancer types mentioned above, another study including 1,044 breast cancer patients, observed that lower preoperative TGs were associated with shorter DFS and worse OS (11). Arthur et al. selected 14,150 men diagnosed with prostate cancer from the Swedish AMORIS cohort who underwent pre-diagnostic measurements of serum glucose, TGs, and TC. The results indicated that neither TC nor TG was associated with death due to cancer (21). Cheng et al. also confirmed that serum lipids were not associated with locoregional recurrence in prostate cancer patients undergoing curative prostatectomy (22). These findings revealed that TG may have different prognostic and predictive values in a variety of cancer settings.

However, few studies have explored the association between TG and esophageal cancer-related deaths. Chen et al. (14) retrospectively analyzed the prognostic value of pretreatment TG in patients with non-ESCC, which accounts for only 10% of all esophageal cancer patients, and multivariate Cox proportional analysis showed that TG was a significant independent prognostic factor in (hazard ratio: 0.49; *p* = 0.009). Unlike Chen’s study, all patients included in this study suffered from ESCC, which accounts for approximately 90% of all esophageal cancers in China. Therefore, our results are more representative and conducive to clinical application in China. In addition, serum TG undergoes dynamic changes during treatment. Therefore, we analyzed the relationship between pre-TG, post-TG, and changes of TG during treatment and survival. We found that pre-TG was not significantly associated with patient outcomes, while post-TG was a strong prognostic factor in both univariate and multivariate analyses of OS in patients with esophageal cancer. Similarly, we also found that the change of TG is a prognostic indicator of survival as a continuous variable in univariate and multivariate analysis. However, the HR and *p* value of TG change are both larger than those of post-TG, so the prediction value of TG change is not as good as that of post-TG. On further analysis, no difference in survival was observed when we divided these patients into two groups (the TG increase group and TG decrease group).

According to the European Society of Cardiology (ESC)/European Atherosclerosis Society (EAS) Guidelines for the Management of Dyslipidemias, hypertriglyceridemia is defined as TGs > 1.7 mmol/L (150 mg/dL) (23). However, this criterion is mainly based on general population, and may be more helpful in guiding the prevention of cardiovascular diseases. The optimal cut-off point which can better predict the survival of cancer patients is unclear, especially ESCC patients. In the present study, we used an outcome-oriented method to determine the optimal cut-off point to maximize log-rank statistics. As a result, we calculated a specific post-TG cut-off (1.47 mmol/L) for patients with ESCC, which is slightly lower than the clinical cut-off point. According to the cut-off of 1.47 mmol/L, the median OS was 31.8 months longer for patients in the high post-TG group than those in the low post-TG group (49.9 months vs. 18.1 months). To further explore the contribution of TG and other influencing factors to survival, we constructed nomograms with a moderate accuracy CI 0.73 (95% confidence interval: 0.71–0.74). In future research, if the sample size can be further improved, it will help to improve the accuracy of the model.

Interestingly, the correlation between serum TG and prognosis seems completely opposite in different tumors as previously mentioned. We hypothesize that this phenomenon is caused by the double-edge effect of TG on cancer patients. Research supporting the association between low TGs and better prognosis argues that hypertriglyceridemia, a metabolic syndrome, may stimulate myeloid-derived suppressor cells (MDSCs), which are ought to be key drivers of immunosuppression and may result in the reduction of both immune surveillance and anticancer cytotoxicity (24). Therefore, hypertriglyceridemia may lead to an immunocompromised status and cancer cell proliferation, thereby worsening survival outcomes (25). He et al. found that increase of triglycerides in ESCC cells may be caused by loss of FBP1, which can promote proliferation, migration, and invasion by regulating fatty acid metabolism (26). However, on the other hand, the serum lipid profile is a biomarker of cancer patients’nutritional status which has a positive impact on survival. Therefore, the impact of TGs on the survival of cancer patients is complicated and specific. Due to the different biological behaviors of different tumors, the positive or negative effects of TG are different. If the positive effect is stronger, it’s a good prognostic factor, and if the negative effect is stronger, it’s a bad prognostic factor.

Specifically, why higher TG could improve survival in ESCC patients? The first potential underlying mechanism may be related to the biological process of serum triglycerides. There are two main sources of serum triglycerides, i.e., decomposition of fat in food and conversion from fructose in the liver. Therefore, a higher serum TG means both better dietary intake and liver metabolic function, which has a significant positive impact on the survival of ESCC patients.

The second possible explanation may be related to the catabolism of triglycerides. Triglycerides, which are composed of three molecules of fatty acids and one molecule of glycerol, are energy rich lipids that can be packaged and exported to peripheral tissues. Generally, they will become a repository of fatty acids and will be broken down according to the needs of the body. The fatty acids separated from triglycerides are free fatty acids, which are efficient heat sources that can be quickly mobilized for life activities. Hence, the high level of serum triglycerides may play the role of energy supplying in the case of insufficient energy intake in patients with esophageal cancer, which is conducive to maintain the patient’s physical condition and survival.

The last reason may be related to the storage of triglycerides, i.e., excessive triglyceride will be stored beneath the skin. One study showed that body fat was significantly associated with TG levels in a nationally representative sample of US adults (27); another research conducted by Zou also showed that there is a significant association between TGs and ectopic fat obesity (28). Subcutaneous fat and visceral fat are important manifestations of patients’ nutritional status, thus a higher TG level indicates that the patient has a better nutritional status (29). Profound loss of adipose tissue is a hallmark of cancer cachexia. Indeed, survival of cancer patients is inversely correlated with severity of cachexia (30). So, unlike the role of a risk factor for cardiovascular disease in healthy people, excessive fat may be a protective factor in cancer patients (31, 32).

Few studies have demonstrated the relationship of body muscle mass and body fat mass with TGs in cancer patients. Clarifying the association of body composition with serum TGs among cancer patients will help clinicians to understand the relationship between lipid metabolism and the nutritional status and prognosis of cancer patients. Okekunle et al. conducted a study that investigated the association between plasma lipids and obesity among breast cancer patients. The results indicated that increasing levels of serum TGs were associated with increasing body mass index among premenopausal breast cancer survivors (33).

Furthermore, we found associations between post-TG and body composition assessment, including muscle area, muscle radiodensity, and adiposity. The post-body mass index, post-subcutaneous adipose tissue area, post-visceral (intra-abdominal) adipose tissue area, post-total adiposity tissue area, and post-total adipose density exhibited a strong positive association with post-TG. However, the mechanism through which body fat mass influences TGs is not completely understood. Individuals with excess central fat tend to show insulin resistance. Further, insulin resistance can promote visceral adipocytes to release excessive free fatty acids; the liver subsequently absorbs these raw materials for the synthesis of TG. Thus, TG may be used as a more economical and convenient indicator of body fat mass.

Nevertheless, the detailed mechanisms of how hypertriglyceridemia may influence cancer survival require further investigation. Moreover, cancer types, treatment periods, such as before and after treatment of the same patient, and the effects of TGs on the prognosis of patients vary; thus, an individualized approach is required.

This study has some limitations. First, other markers of serum lipid profiles, such as high-density lipoprotein cholesterol and low-density lipoprotein cholesterol, were not analyzed in this study. Second, the association between TG and prognosis might be biased by other variables, such as weight gain/loss, treatment patterns, clinical stage, etc. In order to minimize these bias, we have made some efforts, such as using multivariate analysis and multivariate Cox regression models. However, there is still a risk for residual confounding despite conducting multivariable regression analysis. In the next step, we will endeavor to reduce these intervention factors by conducting a prospective, multicenter study with a larger sample size to clarify the correlation between TG and prognosis of esophageal cancer. Third, because all patients included had ESCC, the findings of this study cannot be reliably extrapolated to other populations. Squamous cell carcinoma is the main type of esophageal cancer in China, while adenocarcinoma is the main type in European and American countries. Squamous cell carcinomas and adenocarcinomas have different biological characteristics. The results of this study may thus lack universality. Therefore, further studies that include esophageal cancer patients with different pathological types and ethnicities through international collaborations should be conducted.

## Conclusion

In conclusion, the present study indicates that post-TG could potentially serve as a predictor of OS in ESCC patients. Patients at risk should be followed-up and evaluated more closely, and more clinical trials evaluating the effect of lipid-altering medications on ESCC prognosis should be conducted in the future.

## Data availability statement

The original contributions presented in this study are included in the article/Supplementary material, further inquiries can be directed to the corresponding authors.

## Ethics statement

The studies involving human participants were reviewed and approved by Ethics Committee and the Institutional Review Board of Sichuan Cancer Hospital. The patients/participants provided their written informed consent to participate in this study. Written informed consent was obtained from the individual(s) for the publication of any potentially identifiable images or data included in this article.

## Author contributions

JL, XW, and TL were responsible for conceptualizing and designing this study, data collection, data interpretation, and manuscript drafting. NY and XN played a major role in body composition assessment and data analysis. WG, LX, LL, HB, CL, and HK participated in acquisition of clinical records, data analysis, and revision of the manuscript. All authors read and approved the final version of manuscript.

## Supplementary material

Supplementary Figure 1 The PH assumption tested using Schoenfeld residuals.

Supplementary Figure 2 **(A)** The overall survival (OS) nomogram provides a graphical approach to calculate 2-, 3-, and 4-year OS based on a patient’s combination of clinicopathologic covariates. First, locate the post-triglyceride (TG) and draw a line straight up to the points axis to derive the score associated with post-TG. Repeat for other covariates on the nomogram. Add the scores for each covariate to determine the total score. Draw a vertical line from the total points axis to the 2-, 3-, and 4-year OS to obtain the predicted probability. **(B)** The calibration curves for the nomogram. The x-axis represents the nomogram-predicted probability and y-axis represents the actual probability of esophageal squamous cell carcinoma. KPS, Karnofsky performance score.
