# Supplementary figures and images for: Post-treatment serum triglyceride: An effective biomarker for body fat mass and overall survival in esophageal squamous cell cancer patients treated with chemoradiotherapy
Source: Front Nutr. 2022 Dec 2;9:1050643. doi: 10.3389/fnut.2022.1050643 (PMC9755343; doi:10.3389/fnut.2022.1050643)

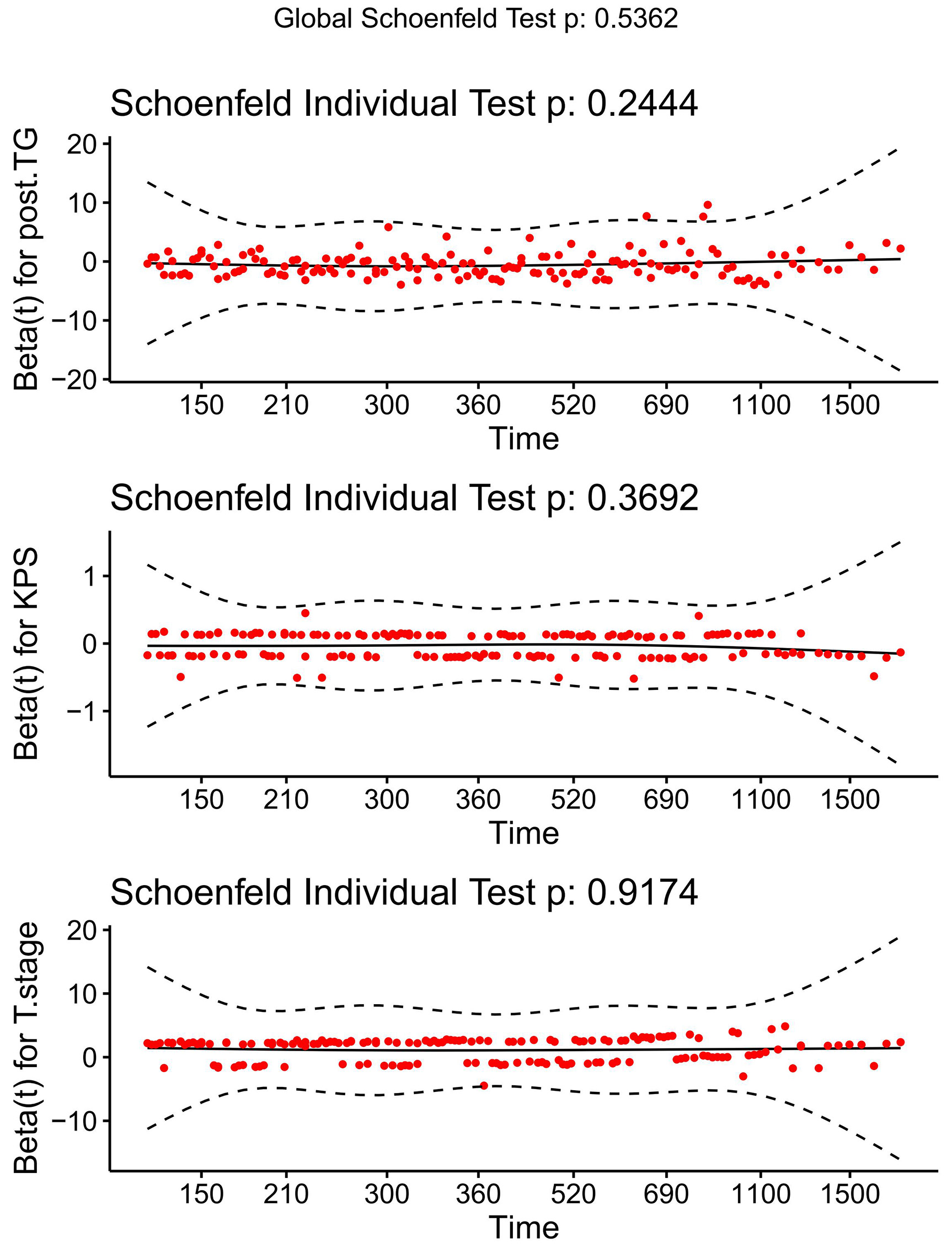

Supplement: Supplementary Figure 1 — The PH assumption tested using Schoenfeld residuals. [file Image_1.JPEG]

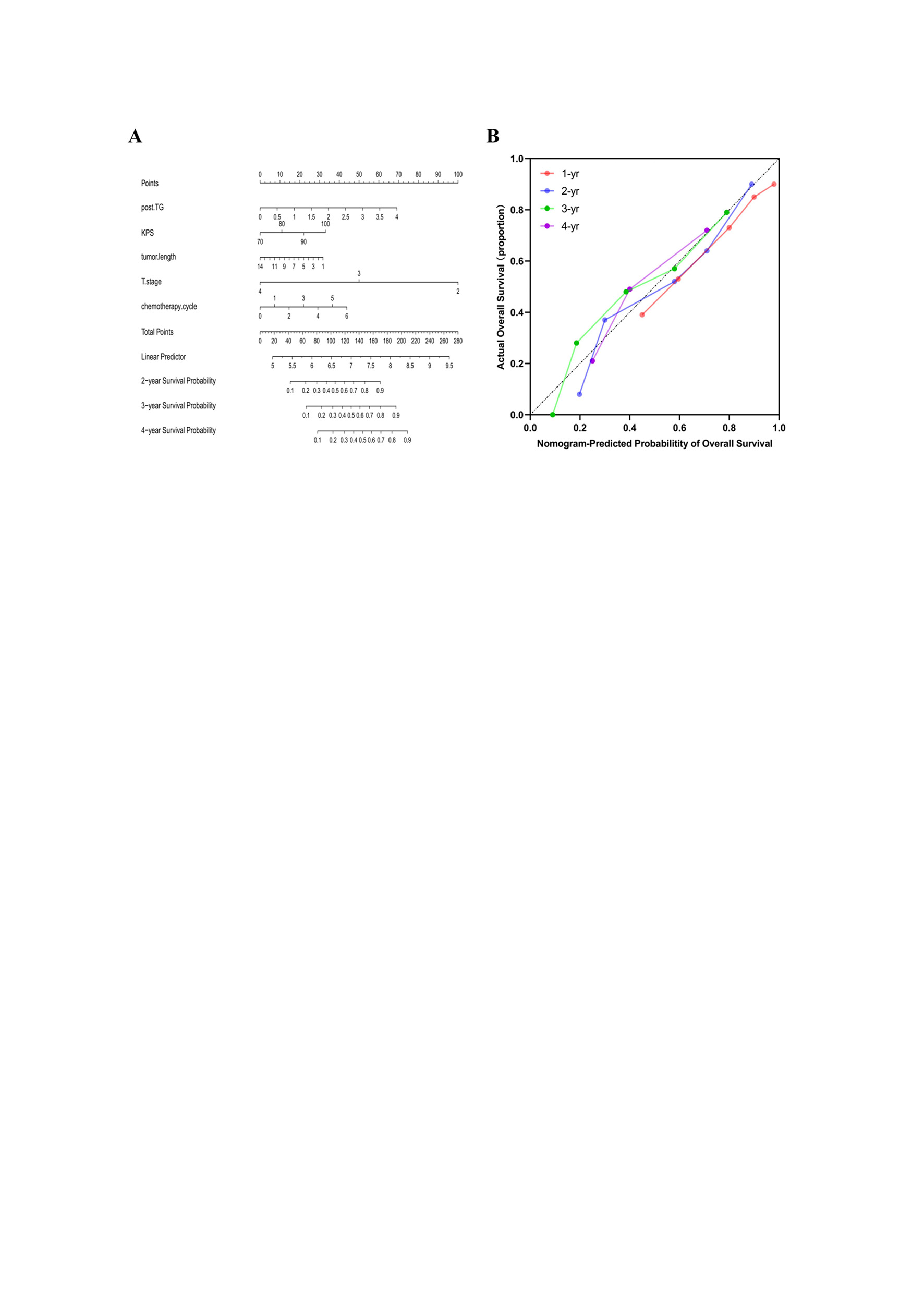

Supplement: Supplementary Figure 2 — (A) The overall survival (OS) nomogram provides a graphical approach to calculate 2-, 3-, and 4-year OS based on a patient’s combination of clinicopathologic covariates. First, locate the post-triglyceride (TG) and draw a line straight up to the points axis to derive the score associated with post-TG. Repeat for other covariates on the nomogram. Add the scores for each covariate to determine the total score. Draw a vertical line from the total points axis to the 2-, 3-, and 4-year OS to obtain the predicted probability. (B) The calibration curves for the nomogram. The x-axis represents the nomogram-predicted probability and y-axis represents the actual probability of esophageal squamous cell carcinoma. KPS, Karnofsky performance score. [file Image_2.JPEG]
