# Supplementary material for: Post-treatment serum triglyceride: An effective biomarker for body fat mass and overall survival in esophageal squamous cell cancer patients treated with chemoradiotherapy
Source: Front Nutr. 2022 Dec 2;9:1050643. doi: 10.3389/fnut.2022.1050643 (PMC9755343; doi:10.3389/fnut.2022.1050643)
Supplement: Supplementary file 3 [file Table_1.docx]

Table S1 Univariate and multivariate Cox regression analyses of change of TG (as a continuous variable) and other factors associated with overall survival

| Characteristics | Total(N) | Univariate analysis | |  | Multivariate analysis | |
| --- | --- | --- | --- | --- | --- | --- |
|  |  | Hazard ratio (95% CI) | P value |  | Hazard ratio (95% CI) | P value |
| change of TG | 257 | 0.69 (0.55-0.87) | **0.002** |  | 0.72 (0.55-0.94) | **0.015** |
| change of TC | 257 | 0.96 (0.82-1.12) | 0.576 |  |  |  |
| change of glucose | 257 | 1.10 (0.98-1.24) | 0.121 |  |  |  |
| Sex | 257 |  |  |  |  |  |
| Female | 57 | Reference |  |  |  |  |
| Male | 200 | 0.99 (0.68-1.45) | 0.985 |  |  |  |
| Age | 257 | 1.01 (0.99-1.03) | 0.230 |  |  |  |
| KPS | 257 | 0.97 (0.95-1.00) | 0.071 |  | 0.96 (0.93-0.99) | **0.008** |
| Smoking history | 257 |  |  |  |  |  |
| No | 110 | Reference |  |  |  |  |
| Yes | 147 | 1.11 (0.81-1.52) | 0.524 |  |  |  |
| Alcohol history | 257 |  |  |  |  |  |
| No | 117 | Reference |  |  |  |  |
| Yes | 140 | 1.28 (0.94-1.75) | 0.122 |  |  |  |
| Tumor location | 257 |  |  |  |  |  |
| cervical | 8 | Reference |  |  |  |  |
| upper thoracic | 53 | 2.19 (0.67-7.16) | 0.196 |  |  |  |
| middle thoracic | 96 | 2.30 (0.72-7.32) | 0.160 |  |  |  |
| lower thoracic | 88 | 2.11 (0.66-6.77) | 0.207 |  |  |  |
| abdominal | 12 | 2.46 (0.67-9.11) | 0.177 |  |  |  |
| Tumor length | 257 | 1.08 (1.02-1.15) | **0.010** |  | 1.07 (1.00-1.14) | **0.044** |
| T stage | 257 |  |  |  |  |  |
| T2 | 29 | Reference |  |  |  |  |
| T3 | 114 | 5.16 (2.07-12.88) | **<0.001** |  | 5.821 (2.31-14.68) | **<0.001** |
| T4 | 114 | 15.45 (6.13-38.93) | **<0.001** |  | 15.03 (5.80-39.00) | **<0.001** |
| N stage | 257 |  |  |  |  |  |
| N0 | 7 | Reference |  |  |  |  |
| N1 | 80 | 6.66 (0.92-48.25) | 0.061 |  | 5.86 (0.79-43.32) | 0.083 |
| N2 | 118 | 6.55 (0.91-47.21) | 0.062 |  | 5.18 (0.71-37.86) | 0.105 |
| N3 | 52 | 15.84 (2.17-115.63) | **0.006** |  | 5.92 (0.78-44.71) | 0.085 |
| Chemotherapy cycle | 257 | 0.83 (0.73-0.94) | **0.003** |  | 0.88 (0.78-1.00) | 0.055 |
| Chemotherapy drugs | 217 |  |  |  |  |  |
| PF | 80 | Reference |  |  |  |  |
| TP | 137 | 0.76 (0.54-1.08) | 0.129 |  |  |  |

KPS, Karnofsky performance score; PF, 5-Fluorouracil + cisplatin; TP, paclitaxel + cisplatin;

TC, total cholesterol; TG, triglyceride;
